# The predictive value of pulmonary function test before transplantation for chronic pulmonary graft-versus-host-disease after allogeneic hematopoietic stem cell transplantation

**DOI:** 10.1186/s12890-022-02278-3

**Published:** 2022-12-12

**Authors:** Lingyi Yang, Jia Cheng, Fei Li, Ruiqi Qian, Xiuqin Zhang, Song Jin, Xuefeng He, Ting Xu, Xiaohui Hu, Xiao Ma, Jia Chen, Yehan Zhu, Feng Chen

**Affiliations:** 1 Department of Pulmonary and Critical Care Medicine, The First Affiliated Hospital of Soochow University, 188, Shizi Street, Suzhou, 215006 Jiangsu Province China; 2 National Clinical Research Center for Hematologic Diseases, Jiangsu Institute of Hematology, The First Affiliated Hospital of Soochow University, 188, Shizi Street, Suzhou, 215006 Jiangsu Province China; 3 Institute of Blood and Marrow Transplantation, Collaborative Innovation Center of Hematology, Soochow University, Suzhou, 215006 China

**Keywords:** Pulmonary chronic graft-versus-host disease, Allogeneic hematopoietic stem cell transplantation, Bronchiolitis obliterans syndrome, Pulmonary function test, The ratio of forced expiratory volume during one second to forced vital capacity, Peak expiratory flow

## Abstract

**Background:**

Pulmonary chronic graft-versus-host disease (cGVHD) after allogeneic hematopoietic stem cell transplantation (allo-HSCT) is a devastating complication and often diagnosed at a late stage when lung dysfunction is irreversible. Identifying patients before transplant who are at risk may offer improved strategies to decrease the mortality. Bronchiolitis obliterans syndrome (BOS) is the typical manifestation of pulmonary cGVHD, which is clinically diagnosed by pulmonary function test (PFT). This study aimed to evaluate the predictive value of PFT pre-HSCT for BOS.

**Methods:**

A single center cohort of 923 allo-HSCT recipients was analyzed, including 15 patients who developed pulmonary cGVHD. Kaplan–Meier method was used to analyze the 3 year progression free survival and 3 year overall survival (OS). A Cox regression model was applied for univariate and multivariate models.

**Results:**

The 3 year cumulative incidence of pulmonary cGVHD was 2.04% (95% CI 1.00–3.08%). According to the cut-off values determined by receiver operator characteristic curve, higher ratio of forced expiratory volume during one second to forced vital capacity (FEV1/FVC) pre-HSCT was correlated to a lower incidence of pulmonary cGVHD [0.91% (95% CI 0.01–1.81%) vs. 3.61% (95% CI 1.30–5.92%), *P* < 0.01], and so as peak expiratory flow to predictive value (PEF/pred) [0.72% (95% CI 0–1.54%) vs. 3.74% (95% CI 1.47–6.01%), *P* < 0.01]. Multivariate analysis showed that FEV1/FVC (HR = 3.383, *P* = 0.047) and PEF/pred (HR = 4.426, *P* = 0.027) were independent risk factors for onset of BOS. Higher FEV1/FVC and PEF/pred level were related to a significantly decreased 3 year non-relapse mortality. The 3 year OS was superior in patients with higher PEF/pred [78.17% (95% CI 74.50–81.84%) vs. 71.14% (95% CI 66.08–76.20%), *P* = 0.01], while FEV1/FVC did not show significance difference.

**Conclusion:**

Our results suggested that PFT parameters such as PEF/pred and FEV1/FVC could be predictors for pulmonary cGVHD and even transplant outcomes before HSCT.

## Backgroud

Allogenic hematopoietic stem cell transplantation (allo-HSCT) has become a well-established life-saving treatment for many patients with hematological malignancies, but post-HSCT complications significantly restrict the prognosis of recipients. Chronic graft-versus-host-disease (cGVHD) is a frequent complication associated with late non-relapse mortality and deteriorated quality of life (QoL) following allo-HSCT [1]. Almost all the organs and tissues can be affected by cGVHD, including lungs which are usually refractory to conventional interventions such as steroids. The pathological aberrant of pulmonary cGVHD is bronchiolitis obliterans (BO), characterized by irreversible fixed airflow obstruction, impaired pulmonary function and a high mortality [2]. Due to the inacceptable risk of lung biopsy for most of HSCT recipients, pulmonary cGVHD is clinically diagnosed with the criteria of bronchiolitis obliterans syndrome (BOS) [3, 4]. Since treatments for established symptomatic BOS are generally ineffective [5], it is essential to identify the patients at risk before HSCT which allows early interventions to decrease the adverse impact of pulmonary cGVHD on prognosis.

Pre-transplant of pulmonary disease is one of the reported risk factors of pulmonary complication post-HSCT [6–10], which is however difficult to measure in practice. Pulmonary function test (PFT) is an efficient quantitative method to evaluate the airway and lung conditions, but the feasibility and accuracy for post-HSCT BOS prediction has not been well validated [8, 11]. Here we conducted a cohort study to favor the identification of high-risk patients.

## Methods

### Study population

This was a retrospective study based on the data derived from the transplant database in our center. The inclusion criteria were: (1) patients who underwent allo-HSCT in our center from 2015 to 2018; (2) patients who performed PFT between last cycle of pre-HSCT treatment and conditioning regimen; (3) patients who had complete information of post-HSCT follow-up based on institutional protocol. The study was approved by the Ethic Committee of our center, and conducted in accordance with Helsinki Declaration.

### Donor and conditioning regimen

Donors of allo-HSCT were selected based on their health, intention, and HLA typing. Hematopoietic stem cells were subcutaneously mobilized by G-CSF (10 mg/kg/d for 5 consecutive days). Both bone marrow (BM) and peripheral blood stem cells (PBSCs) were acceptable as graft. If BM was insufficient, PBSCs was collected on subsequent days.

Myeloablative conditioning regimens were routinely applied in this cohort, including both modified BU/CY and TBI/CY regimen. Modified BU/CY regimen consisted of semustine (Me-CCNU) 250 mg/m^2^ (Day − 10), cytarabine (Ara-C) 2 g/m^2^/q12h (Days − 9 and − 8), busulfan (Bu) 0.8 mg/kg/q6h (Days − 7 to − 5), and cyclophosphamide (CTX) 1.8 g/m^2^/d (Days − 4 and − 3). Modified TBI/CY regimen consisted of: Me-CCNU 250 mg/m^2^ (Day − 8), 12.0 Gy of total body irradiation (TBI) with lung shielding (fractioned administered, Days − 7 and − 6), Ara-C 2 g/m^2^/q12h (Days − 6 and − 5), and CTX 1.8 g/m^2^/d (Days − 4 and − 3). Antithymocyte globulin (ATG) was applied with a dose of 2.5 mg/m^2^/d (Days − 5 and − 2) in unrelated or haplo-identical donor HSCT.

### GVHD management

The GVHD prophylaxis after allo-HSCT routinely consisted of Cyclosporine A(CsA)initiated at 3 mg/kg/d (from Day − 10) and regulated according to plasma concentration, short-term methotrexate(MTX) with 15 mg/kg/d (Day + 1) and 10 mg mg/kg/d (Days + 3,+ 6,+ 11) for HLA identical sibling HSCT. Mycophenolate mofetil (MMF) with 15 mg/kg/d (from Day − 10 to + 60) was combined in unrelated or haplo-identical donor HSCT. Methylprednisolone at a dose of 1–2 mg/kg/d was given immediately as the first-line treatment in case of overt acute GVHD occurrence. The second-line drugs including tacrolimus, anti-CD25 monoclonal antibody, MMF, ATG, etc. The first-line treatment of overt chronic GVHD was steroids and/or CsA.

The diagnosis of pulmonary cGVHD was established according to the NIH criteria [2]: (1) FEV1/FVC < 0.7 or the fifth percentile of predicted; (2) FEV1 < 75% of predicted with > 10% decline over less than 2 years; (3) Absence of infection in the respiratory tract, documented with investigations directed by clinical symptoms, such as chest radiographs, computed tomographic (CT) scans, or microbiologic cultures (sinus aspiration, upper respiratory tract viral screen, sputum culture, bronchoalveolar lavage); and (4) One of the 2 supporting features of BOS: (A) Evidence of air trapping by expiratory CT or small airway thickening or bronchiectasis by high resolution chest CT, or (B) Evidence of air trapping by PFTs: residual volume > 120% of predicted or residual volume/total lung capacity elevated outside the 90% confidence interval. If a patient already carries the diagnosis of chronic GVHD by virtue of organ involvement elsewhere, then only the first 3 criteria above are necessary to document chronic GVHD lung involvement. If BOS is the only clinical manifestation in a patient without a prior established diagnosis of chronic GVHD, a lung biopsy is required.

### Pulmonary function test analysis

All the patients underwent spirometry using a Master screen-PFT system (Jaeger, Germany) according to the American Thoracic Society (ATS) consensus guidelines [12], and the following parameters were measured and analyzed, including the forced expiratory volume during one second (FEV1), forced vital capacity (FVC), the ratio of FEV1/FVC, peak expiratory flow (PEF), maximal expiratory flow at 75% of the FVC has not been exhaled (MEF75), maximal expiratory flow at 50% of the FVC has not been exhaled (MEF50), maximal expiratory flow at 25% of the FVC has not been exhaled (MEF25), maximal mid-expiratory flow (MMEF), and maximal voluntary ventilation (MVV). In order to exclude the effect of some elements, i.e. gender, age, height and weight, all the data were analyzed by the ratio of practical value to predicted value.

### Statistical analysis

In the patients’ baseline information, numerical variables were shown as the mean ± standard deviation (SD) or median with range. Two group comparisons were conducted as independent samples by t-test for those matched to Gaussian distribution, or Kruskal–Wallis test for those did not matched to Gaussian distribution. ANOVA or Kruskal–Wallis tests were applied for multi-group comparisons. Categorical/measurement variables were expressed as the frequency and compared using the chi-squared test. Cumulative incidences or survivals were expressed by probabilities with 95% confidence interval (95% CI). Cut-off value was determined by variables which analyzed by receiver operator characteristic curve (ROC). Kaplan–Meier method was used to analyze the 3 year progression free survival (PFS) and 3 year overall survival (OS). A Cox regression model was applied for univariate and multivariate models. Statistical analysis was conducted using SPSS version24 (SPSS Inc., Chicago, IL, USA). Two-sided *P*-values < 0.05 were considered statistically significant.

## Results

### Baseline characteristics

According to the inclusion criteria, 923 patients were consecutively enrolled in this study. All the patients had a PFT test within 45 days before conditioning regimen. With a median follow-up of 38.4 months, there were 271 patients developing cGVHD according to the NIH 2014 criteria [2], in whom 15 patients had pulmonary involvement.

In order to investigate the clinical and PFT features of pulmonary cGVHD, patients were allocated into three groups: 15 patients who developed pulmonary cGVHD (Group A), 256 patients who developed cGVHD without pulmonary involvement (Group B) and the other 652 patients who had no cGVHD until the end of follow-up (Group C).

The baseline characteristics of the three groups were shown in Table 1, with comparison among the three groups and between Group A and Group B. The ratio of sex, donor type, pre-HSCT pulmonary infection and relapse post-HSCT were comparable among the three groups. Although there were statistical differences in age, underlying diseases and acute GVHD post-HSCT in overall comparison, none of them was validated in the comparison between Group A and Group B except the occurrence of cGVHD. It was suggested that the occurence of cGVHD involving the lungs occurred later than that remitting the lungs (9.1 month versus 5.6 months, *P* = 0.023).

**Table 1:** Baseline characteristics of enrolled patients

Variables	Group A	Group B	Group C	*P* value of overall comparison	*P* value of Group A versus Group B
	Pulmonary cGVHD (n = 15)	Non-pulmonary cGVHD (n = 256)	No cGVHD (n = 652)		
Mean age in years(range)	42 (15–59)	35 (14–67)	38 (11–67)	0.032	0.708
Male, n(%)	10 (66.7%)	157 (62.3%)	379 (58.1%)	0.567	0.679
Underlying disease, n(%)				0.038	1
Leukemia or MDS	15 (100%)	239 (93.36%)	564 (86.50%)		
Lymphoma or myeloma	0	6 (2.34%)	26 (4%)		
Aplastic anemia	0	11 (4.3%)	62 (9.5%)		
Donor type, n(%)				0.109	0.701
Matched sibling	5 (33.3)	71 (27.7)	138 (21.2)		
Matched unrelated	0	22 (8.6)	76 (11.7)		
Haplo-identical	10 (66.7)	163 (63.7)	438 (67.2)		
Pre-HSCT pulmonary infection, n(%)				0.113	1
Fungal infection	1 (6.7)	8 (3.13)	14 (2.15)		
Bacterial infection	0 (0)	3 (1.17)	24 (3.68)		
Other infection	0 (0)	2 (0.78)	6 (0.92)		
Relapse after HSCT, n(%)	2 (13.3)	39 (15.2)	105 (16.1)	0.916	
Onset of aGVHD, n(%)	5 (33.3)	109 (42.6)	220 (33.7)	0.043	0.481
Median of aGVHD post-HSCT, months(range)	0.73 (0.3–3.13)	1.13 (0.3–3.2)	0.9 (0–20.03)	0.043	0.760
Median of cGVHD after HSCT,/months(range)	9.1 (3.1–30.7)	5.6 (0–53.5)	–	0.023	0.023

*MDS* Myelodysplastic syndrome; *MM* Multiple myeloma; *aGVHD* Acute graft-versus-host-disease; *HSCT* Hematopoietic stem cell transplantation

### The impact of pre-HSCT PFT results on pulmonary cGVHD post-HSCT

The Pre-HSCT PFT results were summarized in Table 2. The overall comparison revealed the differences in PEF/pred and MEF75/pred among the three groups, which exhibited an inferior level in pateints developing pulmonary cGVHD. Cut-off values of each PFT parameter to predict pulmonary cGVHD were determined by ROC analyses, and reliable cut-off values were identified in FEV1/FVC (AUC = 0.679, cut-off value = 83.935, specificity = 0.663, sensitivity = 0.692), PEF/pred (AUC = 0.720, cut-off value = 88.25, specificity = 0.627, sensitivity = 0.769), MEF75/pred (AUC = 0.701, cut-off value = 90.35, specificity = 0.663, sensitivity = 0.769), MEF50/pred (AUC = 0.668, cut-off value = 103.65, specificity = 0.343, sensitivity = 1) and MMEF/pred (AUC = 0.670, cut-off value = 83.95, specificity = 0.646, sensitivity = 0.692) with acceptable specificities and sensitivities.

**Table 2:** Comparison of PFT results among the three groups and ROC analyses for pulmonary cGVHD

PFT parameters (median with rage)	Group A	Group B	Group C	*P* value*	ROC^§^	Cut-off value	Specificity (%)	Sensitivity (%)	*P* value of ROC
FVC/pred (%)	94.9 (80.7–109.8)	95.2 (43.7–136.3)	94.1 (38.8–129.3)	0.200	0.554	107.15	0.831	0.385	0.502
FEV1/pred (%)	93.7 (38.3–111.2)	98.5 (18.4–139.9)	97 (29.2–138)	0.544	0.537	82.65	0.847	0.308	0.648
FEV1/FVC (%)	83.82 (38.95–95.6)	86.12 (25.15–99.2)	86.22 (8.01–100)	0.093	0.679	83.935	0.663	0.692	0.027
PEF/pred (%)	83.1 (9.8–110.5)	93.05 (20.8–145.8)	92.9 (9.8–156.3)	0.022	0.72	88.25	0.627	0.769	0.006
MEF75/pred (%)	87.9 (12.6–107.5)	96.35 (5–166.6)	98.4 (4.6–160.1)	0.037	0.701	90.35	0.663	0.769	0.013
MEF50/pred (%)	81 (8.4–103.6)	90.15 (4.2–170.4)	92.6 (8.3–213.3)	0.096	0.668	103.65	0.343	1	0.037
MEF25/pred (%)	77.3 (7.9–101.2)	81 (5.3–201.8)	84.6 (7.3–563)	0.183	0.626	101.25	0.303	1	0.119
MMEF/pred (%)	79.2 (9.1–110.2)	91.55 (4.8–170.4)	92.5 (8.6–202.6)	0.075	0.67	83.95	0.646	0.692	0.035
MVV/pred (%)	94.1 (44.2–101.8)	94.8 (22.9–150.5)	93.5 (0–151.8)	0.103	0.623	101.85	0.321	1	0.143

*pred* Predicted value; *FVC* Forced vital capacity; *FEV1* Forced expiratory volume in the 1st second; *PEF* Peak expiratory flow; *MEF75* Maximal expiratory flow at 75% of the FVC has not been exhaled; *MEF50* Maximal expiratory flow at 50% of the FVC has not been exhaled; *MEF25* Maximal expiratory flow at 25% of the FVC has not been exhaled; *MMEF* Maximal mid-expiratory flow; *MVV* Maximal voluntary volume

*P value refereed to the comparisons of this parameter among three groups

^§^Patients in Group A were defined as positive state in ROC analyses. *P* value of ROC < 0.05 indicated the potential predictive value of the variable for pulmonary cGVHD

Thereafter, pre-HSCT PFT results were transformed into category variables according to the cut-off values. Recognized risk factors for pulmonary cGVHD in univariate analysis included FEV1/FVC, PEF/pre, MEF75/pre and MMEF/pre, while no clinical factor was identified (Table 3). However, multivariate analysis showed that only FEV1/FVC (HR = 3.383, 95% CI 1.02–11.25, *P* = 0.047) and PEF/pre (HR = 4.426, 95% CI 1.19–16.50, *P* = 0.027) were independent risk factor, rather than the parameters reflecting the small airway function.

**Table 3:** Risk analysis for pulmonary cGVHD

	Univariate analysis			Multivariate analysis		
	HR	95% CI	*P* value	HR	95% CI	*P* value
FEV1/FVC	4.642	1.429–15.075	0.011	3.383	1.018–11.245	0.047
PEF/pre	5.816	1.6–21.133	0.007	4.426	1.187–16.499	0.027
MEF 75/pre	6.656	1.832–24.19	0.004			
MEF 50/pre	40.108	0.345–4665.532	0.128			
MMEF/pre	4.106	1.264–13.335	0.019			
MVV/pre	37.115	0.228–6030.347	0.164			
Age	2.958	1.011–8.654	0.048			
Sex	1.454	0.497–4.253	0.495			
Blood type	0.621	0.212–1.818	0.385			
Donor type			0.855			
Sibling versus unrelated	0	0–0	0.981			
Sibling versus haplo-identical	0.736	0.251–2.153	0.576			
Modified Bu/Cy versus modified TBI/Cy	0.045	0–2851.11	0.582			
Onset of acute GVHD	0.923	0.315–2.7	0.883			

*pred* Predicted value; *FVC* Forced vital capacity; *FEV1* Forced expiratory volume in the 1st second; *PEF* Peak expiratory flow; *MEF75* Maximal expiratory flow at 75% of the FVC has not been exhaled; *MEF50* Maximal expiratory flow at 50% of the FVC has not been exhaled; *MEF25* Maximal expiratory flow at 25% of the FVC has not been exhaled; *MMEF* Maximal mid-expiratory flow; *MVV* Maximal voluntary volume

**Fig. 1:**
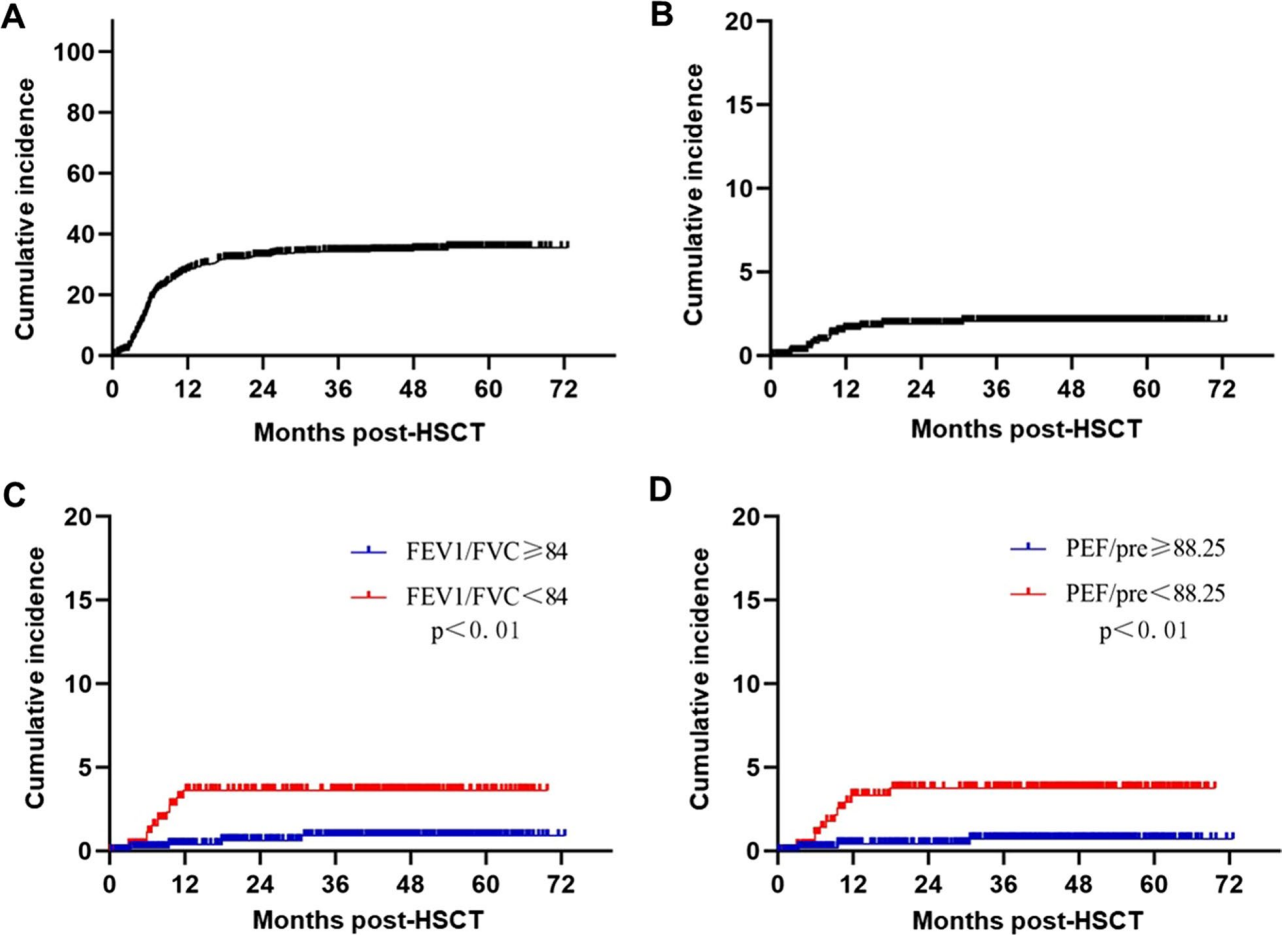
The incidence of cGVHD in the cohort and the impact of PFT results pre-HSCT. **A** Cumulative incidence of cGVHD in the whole cohort; **B** Cumulative incidence of pulmonoary cGVHD in the whole cohort; **C** Comparison of cGVHD incidence between groups with FEV1/FVC level higher or lower than cut-off value; **D** Comparison of cGVHD incidence between groups with PEF/pred level higher or lower than cut-off value

The 3 year cumulative incidence of cGVHD was 34.3% (95% CI 30.89–37.71%) (Fig. 1A), and that of pulmonary cGVHD was 2.04% (95% CI 1.00–3.08%) in the cohort (Fig. 1B). According to the cut-off values, higher FEV1/FVC level pre-HSCT was correlated to a lower incidence of pulmonary cGVHD [0.91% (95% CI 0.01–1.81%) vs. 3.61% (95% CI 1.30–5.92%), *P* < 0.01] (Fig. 1C), and so as PEF/pred level [0.72% (95% CI 0–1.54%) vs. 3.74% (95% CI 1.47–6.01%), *P* < 0.01] (Fig. 1D).

### The effect of PEF and FEV1/FVC before allo-HSCT on transplant outcomes

**Fig. 2:**
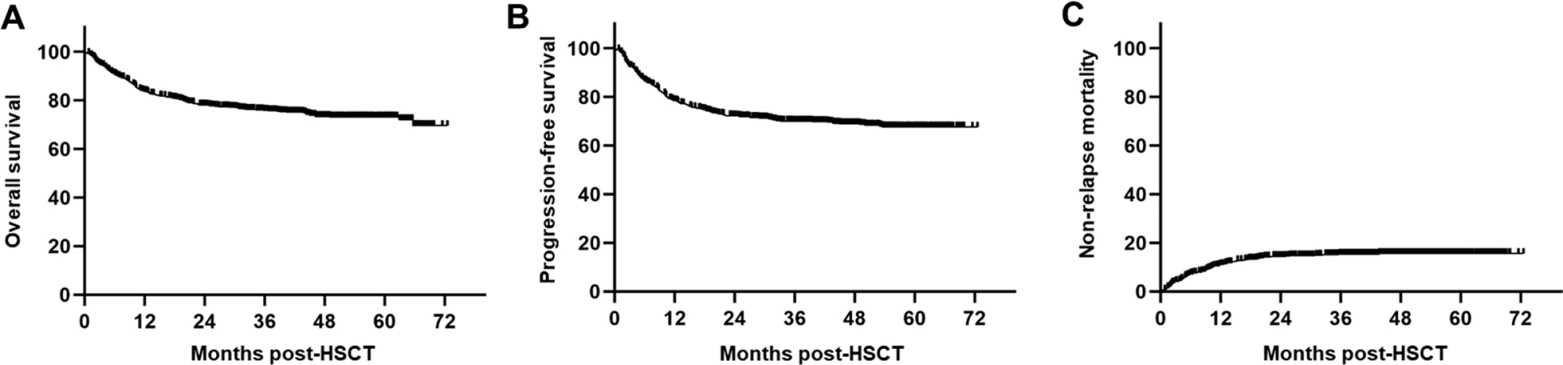
Transplant outcomes of the whole cohort. **A** Overall survival; **B** Progression-free survival; **C** Non-relapse mortality

**Fig. 3:**
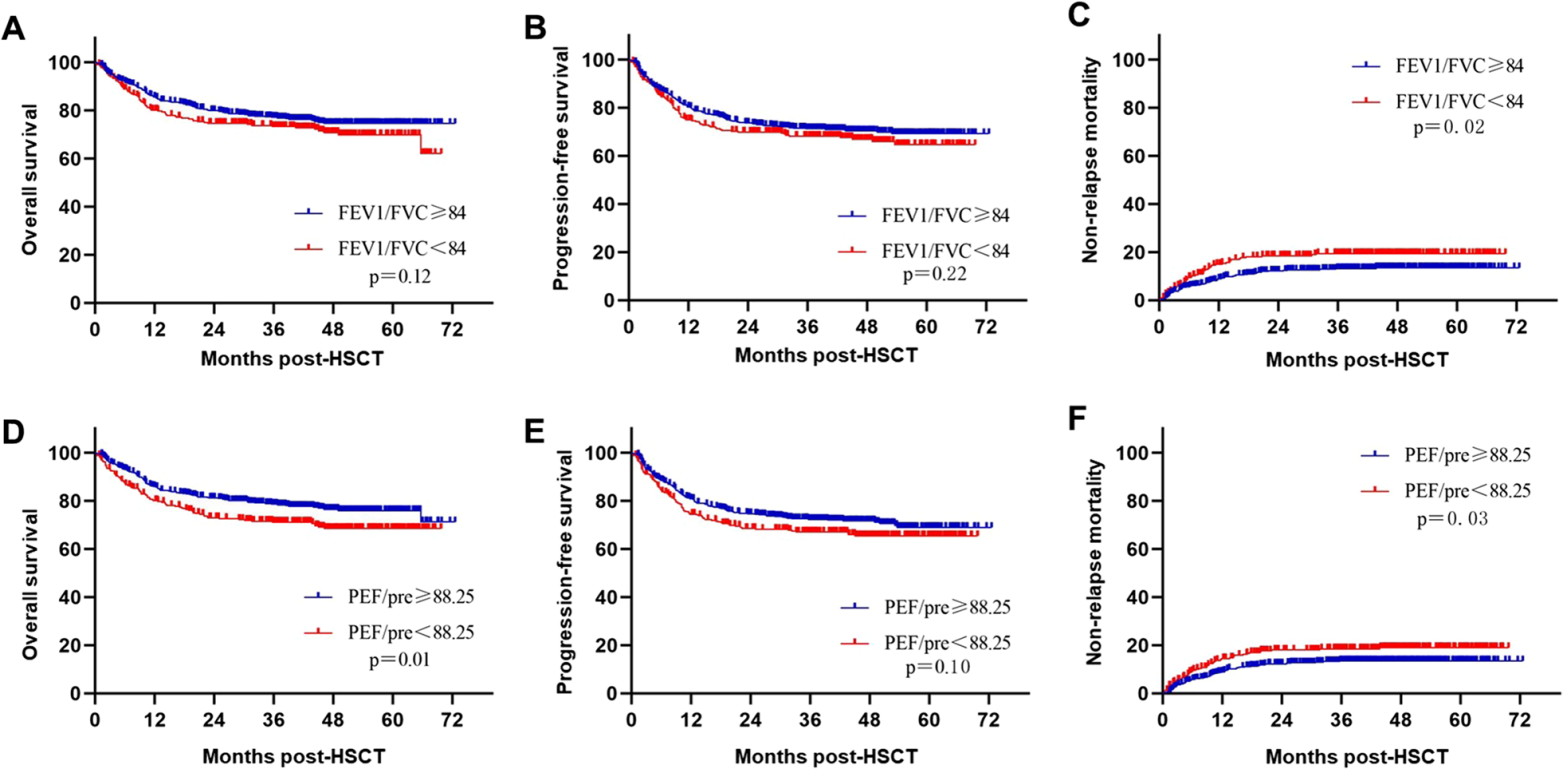
The impact of FEV1/FVC and PEF/pred level pre-HSCT on transplant outcomes. **A** Comparison of overall survival between groups with FEV1/FVC level higher or lower than cut-off value; **B** Comparison of progression-free survival between groups with FEV1/FVC level higher or lower than cut-off value; **C** Comparison of non-relapse mortality between groups with FEV1/FVC level higher or lower than cut-off value; **D** Comparison of overall survival between groups with PEF/pred level higher or lower than cut-off value; **E** Comparison of progression-free survival between groups with PEF/pred level higher or lower than cut-off value; **F** Comparison of non-relapse mortality between groups with PEF/pred level higher or lower than cut-off value

With a median follow-up of 44.5 months for survivors, 3 year overall survival (OS) was 75.60% (95% CI 72.68–78.52%) (Fig. 2A), and 3 year progression-free survival (PFS) was 69.88% (95% CI 66.76–73.00%) (Fig. 2B). The 3 year non-relapse mortality (NRM) was 15.45% (95% CI 12.96–17.94%) (Fig. 2C) in this cohort. Transplant outcomes were compared when stratified by FEV1/FVC and PEF/pred with the cut-off values. It seemed patients with higher FEV1/FVC level pre-HSCT experienced a better 3 year OS [76.71% (95% CI 73.06–80.36%) vs. 73.31% (95% CI 68.14**–**78.48%)] and PFS [71.17% (95% CI 67.33–75.01%) vs. 68.16% (95% CI 62.71–73.61%)] compared to those with lower level of FEV1/FVC, but the differences were insignificant (*P* = 0.12 and 0.22, respectively) (Fig. 3A and B). However, higher FEV1/FVC level was related to a significantly decreased 3 year NRM [13.29% (95% CI 10.35–16.23%) vs. 19.37% (95% CI 14.69–24.05%), *P* = 0.02] (Fig. 3C), which hinted the association between impaired pulmonary function and increased risk of fatal complications post-HSCT.

Of note, PEF/pre level pre-HSCT was a potential predictor for survival. The 3 year OS was superior in patients with PEF/pre ≥ 88.25 to those with PEF/pre < 88.25 with a statistical difference [78.17% (95% CI 74.50–81.84%) vs. 71.14% (95% CI 66.08–76.20%), *P* = 0.01] (Fig. 3D). Besides, higher PEF/pre level was also related to a higher PFS [72.19% (95% CI 68.25–76.13%) vs. 67.06% (95% CI 61.85–72.27%), *P* = 0.10], despite of a marginal significance (Fig. 3E). Meanwhile, the 3 year NRM was 13.69% (95% CI 10.61–16.77%) for patients with statistically higher PEF/pre level versus 18.44% (95% CI 14.05–22.83%) for those with lower PEF/pre level (*P* = 0.03), which was similar to FEV1/FVC (Fig. 3F).

## Discussion

BOS is a severe fatal complication of allo-HSCT, characterized by airflow obstruction on spirometry [13]. It is associated with a significantly increasing NRM for HSCT recipients [13]. The generally poor response to therapy has led to efforts for early identification of high risk patients in whom earlier intervention may prevent irreversible structural damage and ameliorate pulmonary symptoms. Published studies have reported a series of risk factors for pulmonary cGVHD including impaired lung function before and early after HSCT, a myeloablative/busulfan-containing conditioning regimen, cytomegalovirus (CMV) seropositivity, pre-transplant history of pulmonary disease, female donor, unrelated donor and prior acute GVHD [14–16]. However, it remains challenging since most of them are shared with cGVHD involving other organs, and routine medication for allo-HSCT.

Pulmonary function test can not only reflect the respiratory system, but also identify disorders outside the respiratory system, including neuromuscular weakness and cardiovascular processes [17]. The abnormality recognized by spirometry could be separated into obstructive and/or restrictive disorders. Based on the current NIH consensus criteria for cGVHD diagnosis, BOS is a diagnostic and distinct manifestation of cGVHD in the lungs [2]. In the patients who are diagnosed as confirmed BOS, remarkable obstructive dysfunction can be observed, including decreased FEV1/FVC (less than 0.7 or the fifth percentile of predicted), decreased FEV1/pred (less than 75% with more than 10% decline over less than 2 years) [2]. Since PFT is considered to be a non-invasive and attractive strategy to evaluate pulmonary conditions, it is recommended to be taken before and after HSCT dynamically.

PFT before transplantation might be a nonspecific but sensitive indicator of a patient’s general physiological condition, toxicity arising from prior treatment and/or a comorbid illness [19]. Nonetheless, allo-HSCT recipients are generally of good performance status without significant comorbidities, to guarantee the tolerance to the very intensive conditioning chemotherapies and following immunosuppressive periods. Previously reports have shown a significant relationship between PFT prior to allo-HSCT and infectious or noninfectious pulmonary complications post-HSCT, such as respiratory failure [10, 13, 18–21]. However, there is few study concerning the effect of PFT prior to allo-HSCT on the morbidity of BOS. In fact, due to the cautious exclusive criteria for HSCT candidates, it is infrequent for patients eligible for allo-HSCT had significant abnormalities in the PFT before allo-HSCT [6]. Thus, more sensitive parameters and cut-off value should be determined for identifying high risk patients and predicting survival.

The results from the present study have shown that the average values of pulmonary function before allo-HSCT were within the normal range. However, patients developing pulmonary cGVHD had significantly decreased obstructive parameters including PEF/pred and FEV1/FVC when comparing patients without pulmonary cGVHD. These two parameters were independent risk factors for pulmonary cGVHD and NRM after allo-HSCT, and moreover, PEF/pred was a predictor for OS according to our results. The FEV1/FVC is the most important and sensitive parameter in evaluating expiratory flow obstruction [22], and PEF reflects a range of physiological characteristics of the lung elastic recoil, lung volume, large airway calibre, effort, and neuromuscular integrity of individuals [23]. We speculated PEF may be a more comprehensive parameter for the prediction of pulmonary cGVHD, which reflecting not only obstructive factor, but also a variety of integrated factors [23]. Of note, the cut-off values of both PEF/pred and FEV1/FVC were above 80%, which suggested that the normal reference range might not fit for the evaluation HSCT candidates. The validation of applicable cut-off values relies on large scale multicenter studies.

Although in univariate analysis, MMEF/pred which reflected small airway function indicated the onset of pulmonary cGVHD, it was not validated as independent risk in multivariable analysis. MMEF is the most commonly adopted and traditional index of spirometry to assess peripheral airway obstruction [24], which has been described as less effort-dependent than FEV1 [25, 26]. Nakamae et al. [6] previously reported the predictive value of MMEF pre-HSCT for survival post-HSCT. With a cohort of 206 allotransplant recipients, the authors identified MMEF as the most powerful indicator for survival, and furthermore established a pretransplant lung function model involving MMEF. However, the impact of MMEF was not specific on pulmonary cGVHD according to their data. Although MMEF may be a more sensitive marker of small and medium airway obstruction than FEV1, the specificity is relatively low to identify airflow limitation in small airways [27] and its higher coefficient with of variability may restrict its ability [28]. The utility of MMEF independent of FEV1 or FEV1/FVC is still debated [29, 30], while PEF/pred and FEV1/FVC which are significantly correlated with obstructive dysfunction might be stronger indicators for the development of BOS. More cases are required for investigating the correlation between small airway dysfunction and pulmonary cGVHD in stratified analysis in the future.

Parameters related to diffusion capacity are also important components of PFT, but unfortunately not all the patients had relative data in this retrospective cohort. Nevertheless, we also analyzed the impact of diffusion capacity parameters in a subset of our cohort, including the ratio of diffusion capacity for carbon monoxide determined in single breath to predicted value(DLCO SB/pre), the ratio of diffusion capacity for carbon monoxide per liter of alveolar volume to predicted value(DLCO/VA/pre) and the ratio of STPD (standard conditions with temperature 0 °C, pressure 760mmHg and dry) corrected diffusion capacity for carbon monoxide determined in single breath to predicted value(DLCOc SB/pre). All these parameters were comparable among different groups (*P* > 0.05), and none of them was identified by univariate Cox model. We supposed the diffusion capacity parameters had limited effect, since the pulmonary cGVHD predominantly manifested as an obstructive disease.

This study had several limitations, including the inherent shortages of a single-center retrospective study, lack of regular PFT detection post-HSCT, and incomplete information of previous history and therapeutic data. In addition, only 15 patients were diagnosed as pulmonary cGVHD in our cohort, which inevitably impaired the precision of our results. Further studies are warranted to confirm our findings and explore the value of other PFT parameters with larger sample from multicenter cohorts.

## Conclusion

Due to the poor prognosis of pulmonary cGVHD, early recognition of high risk patients is critical for prophylactic or preemptive interventions. PFT is a routinely employed detection before HSCT, which may potentially predict the risk of pulmonary cGVHD and even transplant outcomes according to our data.

## Data Availability

The data can be obtained from the first author Lingyi Yang (yanglingyiruby@163.com), or from the corresponding authors.

## Abbreviations

cGVHD: Chronic graft-versus-host disease
allo-HSCT: Allogeneic hematopoietic stem cell transplantation
HSCT: Hematopoietic stem cell transplantation
PFT: Pulmonary function test
PFS: Progression free survival
OS: Overall survival
ROC: Receiver operator characteristic curve
FEV1: Forced expiratory volume during one second
FVC: Forced vital capacity
PEF: Peak expiratory flow
BO: Bronchiolitis obliterans
BM: Bone marrow
PBSCs: Peripheral blood stem cells
TBI: Total body irradiation
ATG: Antithymocyte globulin
ATS: American Thoracic Society
MEF75: Maximal expiratory flow at 75% of the FVC has not been exhaled
MEF50: Maximal expiratory flow at 50% of the FVC has not been exhaled
MEF25: Maximal expiratory flow at 25% of the FVC has not been exhaled
MMEF: Maximal mid-expiratory flow
MVV: Maximal voluntary ventilation
CMV: Cytomegalovirus
